# Dormancy Signatures and Metastasis in Estrogen Receptor Positive and Negative Breast Cancer

**DOI:** 10.1371/journal.pone.0035569

**Published:** 2012-04-18

**Authors:** Ryung S. Kim, Alvaro Avivar-Valderas, Yeriel Estrada, Paloma Bragado, Maria Soledad Sosa, Julio A. Aguirre-Ghiso, Jeffrey E. Segall

**Affiliations:** 1 Department of Epidemiology and Population Health, Albert Einstein College of Medicine, Bronx, New York, United States of America; 2 Division of Hematology and Oncology, Department of Medicine and Department of Otolaryngology, Tisch Cancer Institute, Mount Sinai School of Medicine, New York, New York, United States of America; 3 Department of Anatomy and Structural Biology and the Gruss Lipper Biophotonics Center, Albert Einstein College of Medicine, Bronx, New York, United States of America; Univesity of Texas Southwestern Medical Center at Dallas, United States of America

## Abstract

Breast cancers can recur after removal of the primary tumor and treatment to eliminate remaining tumor cells. Recurrence may occur after long periods of time during which there are no clinical symptoms. Tumor cell dormancy may explain these prolonged periods of asymptomatic residual disease and treatment resistance. We generated a dormancy gene signature from published experimental models and applied it to both breast cancer cell line expression data as well as four published clinical studies of primary breast cancers. We found that estrogen receptor (ER) positive breast cell lines and primary tumors have significantly higher dormancy signature scores (P<0.0000001) than ER- cell lines and tumors. In addition, a stratified analysis combining all ER+ tumors in four studies indicated 2.1 times higher hazard of recurrence among patients whose tumors had low dormancy scores (LDS) compared to those whose tumors had high dormancy scores (HDS) (p<0.000005). The trend was shown in all four individual studies. Suppression of two dormancy genes, BHLHE41 and NR2F1, resulted in increased *in vivo* growth of ER positive MCF7 cells. The patient data analysis suggests that disseminated ER positive tumor cells carrying a dormancy signature are more likely to undergo prolonged dormancy before resuming metastatic growth. Furthermore, genes identified with this approach might provide insight into the mechanisms of dormancy onset and maintenance as well as dormancy models using human breast cancer cell lines.

## Introduction

The major cause of death from breast cancer is metastasis: the growth of disseminated tumor cells (DTCs) that lodge in distant sites prior to primary tumor surgery. Most successful adjuvant treatments developed to attack DTCs and micrometastases are based on targeting the increased proliferation rate of tumor cells compared to normal cells [1]. Thus, actively proliferating tumor cells are killed or growth-suppressed by adjuvant treatments. However, non-proliferating, dormant DTCs may remain unscathed. Tumor cell dormancy reflects the capability of DTCs or micrometastases to remain at such low numbers that they are undetected for long periods of time [2]–[5]. Modeling of dormancy suggests that this could happen through the induction of quiescence, through balanced proliferation and death due to an impaired angiogenic switch or through immune control [4]. In this report, we test whether the currently available gene expression signatures for dormancy from experimental models that reflect quiescence and angiogenesis regulation could be used to evaluate breast cancer outcome. We find that the dormancy signature is indeed correlated with clinical parameters. Among ER positive tumors, a higher dormancy score is significantly associated with lower hazard of metastasis.

## Results

Models of *in vivo* tumor dormancy driven by tumor cell quiescence [6] or angiogenic failure [7] have identified gene signatures associated with these phenotypes. We hypothesized that these signatures would be helpful in identifying tumors whose disseminated cells would be more prone to undergo dormancy. Based on these expression profiles, we generated a 49-gene signature for tumor cell dormancy (Table 1), in which we consider genes upregulated in dormant cells as positive dormancy genes and genes downregulated in dormant cells as negative dormancy genes. For each gene, we scaled the expression intensities by dividing them by their average intensity across samples. Then we defined the dormancy score (see Materials and Methods) as the difference between the sum of log intensities of the positive dormancy genes and the sum of the log intensities of the negative dormancy genes. All genes were equally weighted in their contributions to the dormancy score. Thus, we set out to determine whether tumors or cell lines that have a higher dormancy score showed any association with clinico-pathological parameters.

**Table 1:** Dormancy signature genes.

Status in Dormant Cells	Gene Symbols
Up-regulated	ACVR1, ADAM10, AMOT, BHLHE41, COL1A1, COL4A5, CTSD, DDR1, EPHA5, GATA6, HIST1H2BK, IGFBP5, MMP2, NR2F1, P4HA1, SOX9, SREBF1, STAT3, TGFB2, THBS1, TP53, TPM1
Down-regulated	APEX1, ASNS, ATF3, ATF4, BUB1, BUB1B, CDKN3, CEBPG, CKS2, DNMT1, DTYMK, EGFR, EGR1, ESM1, FOSL1, FOXD1, FOXM1, IGF1R, IL8, JUN, MMP1, NT5E, ODC1, PIK3CB, PLAT, TIMP3, TK1

**Figure 1:**
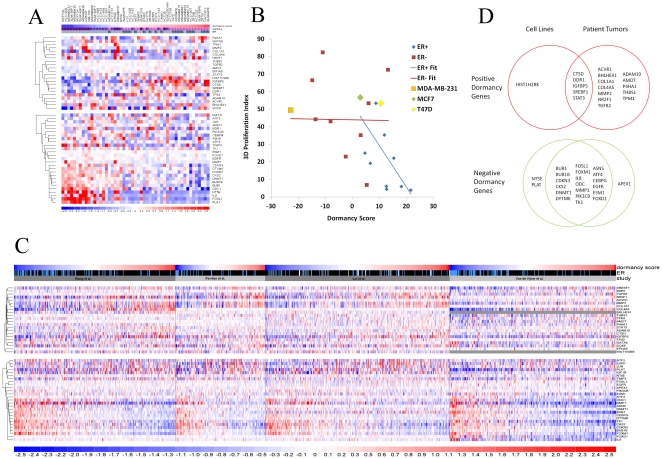
Clustering of dormancy signature scores. A) Dormancy score analysis in breast cancer cell lines. The cell lines are ordered by dormancy scores (low to high from left to right). The rows correspond to genes and the columns represent cell lines. Expression levels for positive dormancy genes (upregulated genes - top section) and negative dormancy genes (downregulated genes - bottom section) were clustered by a hierarchical clustering algorithm. The colors represent log_2_ fold change compared to the average from −2.5 (blue, below average) to +2.5 (red, above average) with white as the average value. A, B, and L stand for Basal A, Basal B, and Luminal classifications, respectively. B) Correlation of cell line dormancy scores with proliferation indices from Table 1 of [9] with ER- lines plotted as squares and ER+ lines plotted as diamonds. Straight line fits of ER- (blue, Spearman correlation coefficient r = .027) and ER+ (red, r = −0.76) cell lines are plotted. The ER status of HCC1500 is unclear (ATCC indicates it as ER+ while it is ER- by gene expression and Western blot in [8]) and it was not used in the analysis. MDA-MB-231 (large orange square), MCF7 (large green diamond) and T47D (large yellow diamond) are identified. C) Patient tumor analysis. The four clinical studies were clustered as in A. In the ER status bar, ER status is indicated by black (ER+), blue (ER−) or white (not determined) bars. The two genes for which probes were not present in the van de Vijver et al. data set are represented by gray bars. D) Comparison of clustering of cell lines and patient data. Top: Positive dormancy genes that are upregulated in high dormancy score cell lines or patients. Bottom: Negative dormancy genes that are up regulated in low dormancy score cell lines or patients.

We first applied the dormancy score to published microarray data of 51 breast cancer cell lines grown in tissue culture [8]. We found that ER positive breast cancer cell lines have significantly higher dormancy scores than ER negative ones (p<0.0001; Mann-Whitney test). As a general trend, as the dormancy score increases, the cell line type changes from basal type B to basal type A to luminal (Figure 1A). A cluster of 6 positive dormancy genes (STAT3, HIST1H2BK, CTSD, SREBF1, IGFBP5 and DDR1) is more highly expressed in lines with higher dormancy scores. Conversely, a larger cluster of negative dormancy genes is upregulated in lines with lower dormancy scores. Included in this cluster are pro-proliferative genes (NT5E, IL8, PLAT, FOSL1, ODC1). The expression profiles of genes positively correlated with dormancy tended to be less homogeneous compared that of the genes inversely correlated with dormancy (Figure 1A). This might be because these expression profiles were obtained from breast cancer cells proliferating in culture while dormancy is mediated by a G0−G1 arrest not achieved in these conditions. Thus, the positive dormancy genes might become more synchronous in their expression *in vivo*.

To evaluate *in vivo* dormancy properties of these subgroups, GFP labeled MDA-MB-231, MCF7, and T47D cells were labeled with Cell Trace Violet and injected into the mammary fat pads of SCID mice. After 3 days, the retained label and proportions of GFP positive cells were determined by FACS. Label retention was positively correlated with dormancy signature score (Spearman correlation coefficient +0.71, p<.01). The proportion of GFP labeled cells in the mammary fat pad was inversely correlated with dormancy signature score (Spearman coefficient −.857, p<.001), consistent with increased dormancy. We next determined if there was any association between the dormancy score and the inherent proliferative capacity of these cancer cell lines. To this end we compared the dormancy score with published proliferation indices measured in 3D growth conditions available for 22 of the cell lines [9]. We found that there was no statistically significant correlation between proliferation index and dormancy score for ER- cell lines (p>.9), but a significant correlation for ER+ cell lines (Spearman correlation coefficient −0.76, p<.01, Figure 1B). Thus, the dormancy score is indicative of a program that imposes slower growing kinetics on ER+ tumor cells in 3D culture, suggesting that it might be indicative of slow proliferation or quiescence in vivo.

To test if the in vivo conditions in patients reveal a similar or better relationship between the dormancy scores and breast cancer progression, we evaluated the dormancy signatures of clinical breast cancer samples. We used four published microarray data sets that included well annotated invasive breast cancers with at least seven years of follow-up [8], [10]–[13]. We performed an analysis of all the samples in the four studies, stratified by study, and found that the dormancy scores were significantly higher in ER+ tumors compared to ER- tumors (p<0.0000001; stratified Mann-Whitney rank sum test, Table 2). This is consistent with our analysis of the breast cancer cell lines. Analysis of the individual studies also showed a significantly higher dormancy score in ER+ vs. ER- tumors in three out of four (p<0.001) while one study [10] showed a weak trend in the same direction (Table 2). Thus, although the genes selected for the dormancy score were identified from gene expression patterns of cell lines grown in tissue culture, their predictive value for an *in vivo* phenotype in the experimental models [6], [7] could be extended to differentiating between primary tumors with different ER status.

**Table 2:** Dormancy scores of ER positive and ER negative tumors.

Study	P value*	P value from stratified test#
Van de Vijver et al	<0.00001	P<0.0000001 (Z = 9.2)
Wang et al.	<0.00001	
Pawitan et al.	<0.001	
Loi et al.	0.82	

* The statistical significance of the difference in dormancy score between ER+ and ER− tumors was determined using the Mann-Whitney rank sum test.

# Mann-Whitney rank sum test (van Elteren's test) stratified by studies using all samples from 4 studies.

Examination of the clustering of the clinical samples revealed a difference between the positive and negative dormancy genes (Figure 1C). The set of negative dormancy genes that was upregulated in the tumors with low dormancy scores was similar to the set that we observed in the cell lines; of the 22 negative dormancy genes that were upregulated in the cell lines with low dormancy scores, 19 were also upregulated in tumors with low dormancy scores, and one additional gene was upregulated in the tumors with low dormancy scores (APEX1). However, there was a more dramatic change in the positive dormancy genes. As noted above, in the cell lines with high dormancy scores there were fewer positive dormancy genes that were upregulated (6), and of those 5 were also upregulated in tumors with high dormancy scores. However, in patient tumors with high dormancy scores there were an additional 12 positive dormancy genes that were upregulated (Figure 1D). This indicates that in vivo the positive dormancy genes are more synchronously expressed and this may prime these cells upon dissemination to fully enter a G0–G1 arrest in secondary sites.

**Figure 2:**
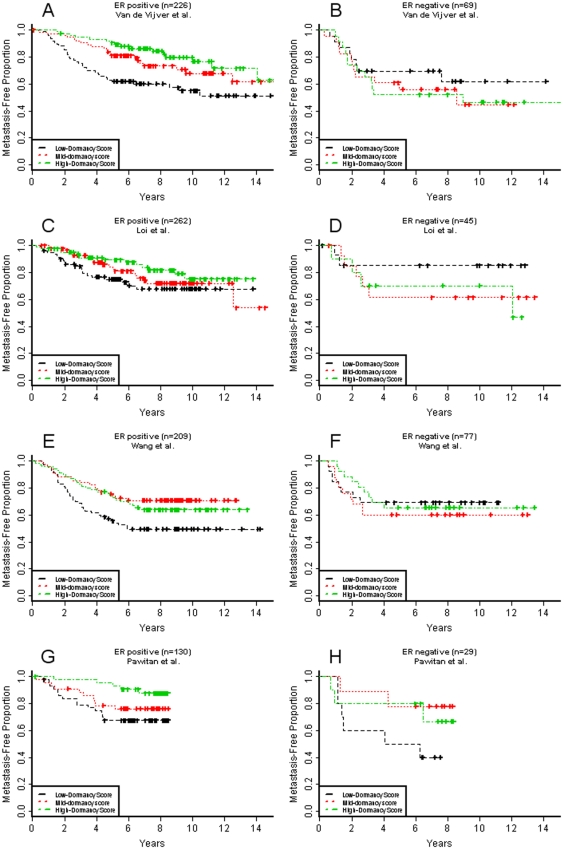
Metastasis-free analysis for four clinical studies. (A, B) van de Vijver et al., (C, D) Loi et al., (E,F) Wang et al., (G, H) Pawitan et al. Kaplan Meier estimates of metastasis-free proportion among patients with high (upper third, green), medium (middle third, red), and low (bottom third, black) dormancy scores for patients with ER+ (A,C,E,F) and ER− (B,D,F,G) tumors.

We then tested the association between dormancy and distant metastasis free survival. Time to metastasis was determined as a measure of the persistence of asymptomatic and possibly dormant disease. In all four studies, patients whose ER+ tumors had higher dormancy scores showed significantly or moderately significantly reduced rate of recurrence (Table 3). A stratified proportional hazards model combining all ER+ tumors in the four studies indicated 2.1 times higher hazard of metastasis among patients with LDS tumors compared to those with HDS tumors (p<0.000005). Kaplan Meier plots confirmed the correlation of dormancy score with survival for ER+ tumors but not for ER- tumors (Figure 2).

**Table 3:** Hazard ratios of metastasis for ER+ tumors according to dormancy score.

Study	P value*	Hazard Ratio**	95% confidence interval	P-value from stratified analysis#
Van de Vijver et al.	0.001	2.62	(1.44, 4.77)	P<0.000005
Pawitan et al.	0.014	3.31	(1.19, 9.20)	
Wang et al.	0.052	1.66	(0.99, 2.77)	
Loi et al.	0.050	1.94	(0.99, 3.80)	

* The metastasis-free survival times of ER + tumors in high dormancy score and low dormancy score groups were compared using Cox's proportional hazard model.

** Hazard of metastasis among patients with low dormancy score relative to those with high dormancy score.

# Cox's PH analysis stratified by studies using all samples from four studies.

The data gathered in Figures 1and 2 revealed that ER+ luminal type tumor cells carry a dormancy gene signature. We hypothesized that some of these genes might contribute to dormancy of ER+ tumor cells. We have previously shown that both BHLHE41 and NR2F1 are required for or associated with dormancy of squamous carcinoma cells [6]. Thus, we tested in the ER+ luminal type breast cancer cell line MCF-7 whether knocking down these genes affected latency and subsequent tumor growth. MCF-7 is known to show long latency periods before achieving a high rate of tumor take [14]. We hypothesized that if these genes were required to maintain dormancy then this phase should be shortened and/or the tumor-take enhanced.

**Figure 3:**
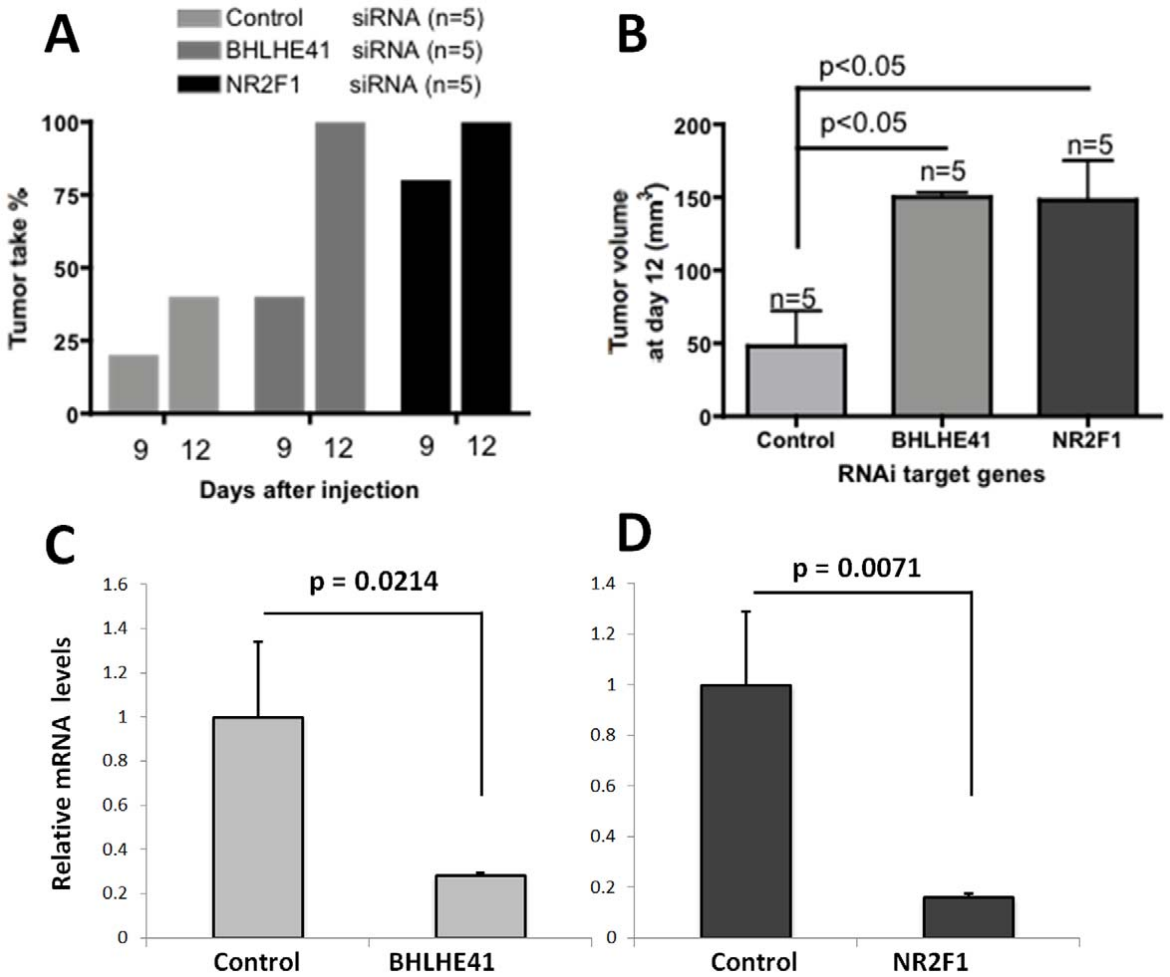
RNAi suppression of dormancy upregulated genes accelerates tumor take and growth of ER+ luminal MCF-7 cells. (A) Percent of tumor take at the indicated time points after injecting with 4 10^6^ MCF7 cells treated with the indicated siRNAs in the mouse mammary fat pad. (B) Tumor volume (mm^3^) at 12 days for tumors generated by MCF7 cells treated with the indicated siRNAs and injected in the mouse mammary fat pad. (C–D) Q-PCR analysis for the expression of BHLHE41 (C) and NR2F1 (D) mRNAs after 48 hrs of treatment with control or specific siRNAs targeting these mRNAs.

MCF-7 cells were transfected for 24 hrs with a control scrambled siRNA or siRNAs targeting NR2F1 or BHLHE41, then injected into the mammary fat pads of female NSG mice (Figure 3A–B) with a fraction of the cells used to evaluate mRNA knock down using qPCR (Figure 3C and D). The siRNAs caused a>75% reduction in BHLHE41 or NR2F1 mRNA normalized to GAPDH (Figure 3C and D). Three days after injection in the fat pad and thereafter the mice were monitored for tumor take. We found that 12 days after tumor cell injection only 40% of the mice injected with control siRNA-treated cells had palpable tumors, while palpable tumors were present in 100% of the mice injected with BHLHE41 or NR2F1 siRNA treated cells (Figure 3A–B). Furthermore, the tumors produced by BHLHE41 and NR2F1 siRNA treated MCF-7 cells appeared to proliferate faster; the final tumor volume at day 12 was significantly larger for tumors produced by BHLHE41 and NR2F1 siRNA treated cells compared to those produced by control siRNA treated cells (Figure 3A–B). These results demonstrate that the genes identified in the dormancy signature are functional in ER+ luminal breast cancer cell lines in limiting tumor growth, possibly through the induction of a dormant phenotype.

## Discussion

We present a dormancy score based on gene signatures developed by combining dormancy expression profiles from a variety of cancer types using even-weighting of all the genes. Although neither recurrence information nor ER status was used to select these genes or to refine the scores, we found that luminal, ER+ breast cancers were more likely to have a high dormancy score. Positive dormancy genes were more synchronously upregulated in patient tumors than in cell lines grown *in vitro*, demonstrating the importance of the tumor microenvironment on dormancy properties. Furthermore, the rate of recurrence was significantly reduced for patients whose tumors were ER+ and had a high dormancy score. These results are consistent with the observed clinical outcome that ER+ tumors tend to recur later than ER- tumors [15]. Furthermore, since the analysis utilized data from primary tumors, which in some cases came from in patients that underwent mastectomy, the findings suggest that DTCs coming from ER+ tumors with a high dormancy score are more likely to enter a dormancy state.

We found a correlation between published data on 3D in vitro proliferation indices of ER+ cell lines and dormancy score, with increased dormancy score correlating with reduced 3D proliferation index. However, there was no statistically significant correlation between 3D proliferation index and dormancy score for ER- cell lines. This parallels the patient data, in which the dormancy signature inversely correlates with rate of recurrence for patients with ER+ tumors but not for patients with ER- tumors. This raises the possibility that 3D proliferation index could be used as a surrogate in vitro assay to explore dormancy mechanisms in ER+ tumors, which would then be validated with in vivo assays. It also suggests that dormancy in vivo could reflect a reduced proliferation rate, as evidenced by the frequent lack of proliferation markers in DTCs (Aguirre-Ghiso, 2007). This slow proliferation or quiescence phenotype could explain the presence of non-productive residual disease by limiting the disseminated tumor cell population to a steady state level, potentially complemented by immune attack and limited angiogenesis. Comparing the dormancy scores of luminal A vs luminal B tumors (which have a higher proliferation index [16]) in the van de Vijver and Pawitan datasets is consistent with this possibility: the median dormancy score is significantly lower in the luminal B tumors compared to the luminal A tumors (van de Vijver: p<10^−4^ and Pawitan: p<10^−5^). Intriguingly, in a meta-analysis of all luminal B tumors from the two datasets, we found 2.6 times lower hazard of metastasis among patients with high dormancy score tumors compared to those with low dormancy scores (p<.04), indicating that the dormancy score has potential value for differentiating between patients within this subtype of tumors.

The dormancy signature also incorporates elements of p38 signaling [6], and p38-induced quiescence combined with reduced ability to undergo an angiogenic switch might contribute to ER+ tumor cell dormancy. Several genes in the dormancy signature derived from quiescent cells [6] are regulators of angiogenesis. For example, THBS1 is an angiogenesis inhibitor induced by p38 [6], [17], [18]. It is possible that emergence from prolonged quiescence requires the immediate recruitment of blood vessels to support nascent tumor expansion and that might explain why anti-angiogenic genes are embedded in the quiescence signature. Genes such as BHLHE41 (also referred to as BHLHB3 or Sharp-1), NR2F1 and p53 have been linked to the induction of quiescence in an experimental system [6]. Furthermore, BHLHE41 was independently discovered as a metastasis suppressor gene in breast cancer [19]. This suggests that these genes might be prevalent transcription factors required for persistent induction of dormancy to suppress metastatic growth. This is further supported by our findings that BHLHE41 or NR2F1 knock down strongly stimulated tumor take and growth of MCF-7 ER+ luminal cancer cell lines during the initial dormancy phase that precedes logarithmic tumor growth.

It is interesting to speculate that dormancy signatures might be developed for other breast cancer subtypes such as ER- tumors, and that the genes involved would presumably reflect subtype-specific quiescence mechanisms. Targeted treatments that lead to maintenance of the activity of genes that induce dormancy in a breast cancer subtype might be useful in prolonging quiescence, keeping the disease in a chronic state. Alternatively, inhibition of key survival genes in subtype-specific pathways could attack dormant tumor cells before they resume growth and progression, thereby reducing the risk of recurrence.

## Materials and Methods

### Dataset sources

We downloaded expression data set of 51 breast cancer cell lines and ER status of the cell lines from Neve et al. [8], in which expression profiles were measured using Affymetrix HG-U133A arrays. We downloaded expression data sets of human breast tumors published by Pawitan et al. ([12], n = 159), Loi et al ([10] n = 327), Wang et al. ([11], n = 286), and van de Vijver et al. ([13] n = 295). Loi et al, Wang et al, and Pawitan et al. measured expression using Affymetrix HG-U133A and HG-U133B arrays. Van de Vijver et al. measured expression using Agilent cDNA arrays. Dr. Y. Pawitan kindly provided the ER status of the samples in Pawitan et al.

### Preprocessing of microarray data

For the tumor samples from Pawitan et al., Loi et al., and Wang et al., we obtained CEL files. The DNA-Chip Analyzer was used to normalize all CEL files to the baseline arrays and compute the model-based expression (PM-only model; [20]). For the cell lines in Neve et al., we obtained already preprocessed data: the published intensities were in log_2_ scale and we transformed them to linear values before computing the dormancy scores.

For the tumor samples from van de Vijver et al. [13], the data are ratios between the intensity of individual samples and the average intensity from a cy-labeled cRNA from individual tumor mixed with the same amount of reverse-color cy-labeled pool that consisted of an equal amount of cRNA from each patient. Published ratios of intensities were in log_10_ scale and we transformed them to linear values before computing the dormancy scores.

For multiple Affymetrix probe sets that targeted a common gene, we used the ‘_at’ probe set with the highest variation measured by the coefficient of variation. When there were no ‘_at’ probe sets targeting a gene, we retained the ‘_s_at’ probe set with the highest variation. When neither of those probe set types were present for a gene, we retained the probe set with the highest variation.

### Computation of dormancy scores

We defined dormancy score for the i^th^ sample of each data set as following:
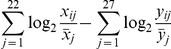



where x_i1_, …, x_i22_ are intensities of the 22 up-regulated genes, 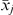 is the average intensity of j^th^ up-regulated gene across all samples, y_i1_, …, y_i27_ are intensities of the 27 down-regulated genes, and 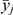 is the average intensity of j^th^ down-regulated gene across all samples (Table 1). In van de Vijver et al., the arguments for the logarithmic functions are directly measured for each gene: the ratio between the intensity of a sample and the average intensity across all samples is measured by the relative brightness of cy-labeled cRNA and reverse-color cy-labeled pool (See Preprocessing of microarray data). For all other data sets, we computed the ratios by averaging the intensities across samples. There were no probes for BHLHE41 or HIST1H2BK in the van de Vijver data set and thus the calculation of the dormancy score for that data set did not include those genes, all other data sets included probes for all the dormancy signature genes.

### Statistical Analyses

We performed the Mann-Whitney test [21] to compare dormancy scores of ER+ versus ER- cell lines or tumors in each study For analyses pooling samples from all four clinical studies, we performed the stratified Mann-Whitney test (or van Elteren's test, [19]),stratifying by study.

In addition, for each clinical study, we tested the association between dormancy and the hazard of distant metastasis by first separating tumors by dormancy score into three equally sized groups: tumors with High/Mid/Low Dormancy scores, referred to as HDS, MDS, or LDS, respectively. Then we tested if the hazards of metastasis were different for the HDS group and LDS groups by fitting a Cox proportional hazards model [22] using samples in all three groups. Kaplan-Meier estimates of relapse-free survival were computed and plotted for 6 groups: ER+ patients with high dormancy scores (HDS), ER + patients with MDS, ER+ patients with LDS, ER- patients with HDS, ER- patients with MDS, and ER- patients with LDS. For analysis pooling all samples from four clinical studies, we performed Cox's proportional hazards regression analysis stratified by studies.

We used time-to-distant metastasis in Loi et al. and van de Vijver et al. as the outcome. In Wang et al. and Pawitan et al., we used time-to-relapse (local or distant) as outcome because it was the only available recurrence measurement in their studies. All statistical analyses were performed using the R statistical environment [23]. The code and data files used for analysis are available as supporting information (Text S1, Dataset S1, Dataset S2, Dataset S3, Dataset S4, Dataset S5 and Dataset S6).

### Xenograft studies

The experiments involving animals were performed according to standards approved by the IACUC of Albert Einstein College of Medicine and Mount Sinai School of Medicine. For comparing MCF7, MDA-MB-231 and T47D lines in vivo, lines were grown in DMEM (Life Technologies) supplemented with 10% FBS (Atlanta Biologicals) and (Gemini Bio-Products), FBS, and 0.5% penicillin/streptomycin (Invitrogen). Cells were labeled with 5 uM CellTrace Violet (Invitrogen) for 20 minutes and then cultured overnight. The next day, cells were trypsinized, centrifuged to remove trypsin, resuspended in PBS and diluted 1∶1 with Matrigel to a final concentration of 5 million/ml. 0.1 ml was injected into the mammary fat pad of 6–8 week old female SCID mice. For T47D and MCF7 injections, an estrogen pellet (0.36 mg, 60 day release, Innovative Research of America) was inserted subcutaneously the day before injection of tumor cells. Cell Trace labeling was quantitated using FACS analysis using Rainbow standards. After 3 days, the animals were euthanized, the mammary fat pad was dissected out, chopped into fine pieces, and dissociated in PBS with collagenase IV (final concentration of 6 mg/ml, C5138, Sigma), hyaluronidase (final concentration of 1 mg/ml, H3506, Sigma), and DNase I (final concentration of 0.25 mg/ml, D5025-15KU, Sigma) for 30 minutes with continuous agitation at 37 degrees Celsius. Following digestion, samples were washed twice in sterile PBS, filtered for single cells, and analyzed by flow cytometry for level of Cell Trace labeling of tumor cells and number of tumor cells using the GFP labeling to identify tumor cells.

For siRNA experiments, MCF-7 cells were cultured on DMEM medium (Cellgro) supplemented with 10% FBS (HyClone) and 1% Penicillin/Streptomycin (Gibco). After reaching 80% confluence, cultures were transfected with siRNAs targeting BHLHE41 (Santa Cruz), NR2F1 (Ambion) or control scrambled siRNA (Ambion) at a final concentration of 80 nM using Lipofectamine™ RNAiMAX (Invitrogen) and following the manufacturer instructions. A second transfection was performed after 24 hours to achieve an efficient knockdown. siRNA knockdown was analyzed by qPCR using iQ SYBR® Green Supermix (BioRad). 48 h post-transfection cells were washed, detached and resuspended on PBS+/+ at a concentration of 4×10^6^ cells/70 µL PBS+/+. Prior to injection, 70 µL of Matrigel (Becton Dickinson) was added to the mix and a total of 140 µL was injected into the left mammary fat pad of female NSG mice (n = 15). Tumors were monitored 3 days after injection and thereafter for tumor take. Palpable tumors were measured and size was calculated following the equation [(length×width^2^)/2] = tumor volume (mm^3^).

### Quantitative PCR analysis

RNA was isolated from MCF-7 cells with TRIzol® reagent following the manufacturer's indications (Invitrogen). Reverse transcription was performed using M-MuLV Reverse Transcriptase (New England Biolabs) and quantitative PCR was performed on a CFX 96™ Real Time System (BIORAD) using iQ SYBR® Green Supermix (Invitrogen) using normalization to GAPDH. The human forward and reverse primer sequences used were: BHLHE41, 5′- CTGATGCTGTTGCTCGGTTA -3′ and 5′- TGCAGACTCTGGGACATCTG -3′, NR2F1, 5′- GCCTCAAAGCCATCGTGCTG -3′ and 5′- CCTCACGTACTCCTCCAGTG -3′, GAPDH 5′-CCCCTGGCCAAGGTCATCCA-3′ and 5′- ACAGCCTTGGCAGCGCCAGT-3′. Statistical analysis was performed with GraphPad Prism 5.0 (San Diego, CA) and *p* values were calculated using one-way ANOVA followed by the Bonferroni multiple comparison post test or the unpaired *t* test.

## Supporting Information

Text S1 R code that describes the analyses performed with the supporting information data files.
(PDF)

Data S1 Data set referred to in Text S1 as dt.Loi.signature.xls.
(XLS)

Data S2 Data set referred to in Text S1 as dt.Neve.signature.xls.
(XLS)

Data S3 Data set referred to in Text S1 as dt.NKI295.signature.xls.
(XLS)

Data S4 Data set referred to in Text S1 as dt.Pawitan.signature.xls.
(XLS)

Data S5 Data set referred to in Text S1 as dt.Wang.signature.xls.
(XLS)

Data S6 Data set referred to in Text S1 as si.xls.
(XLS)
