# Supplementary material for: Dormancy Signatures and Metastasis in Estrogen Receptor Positive and Negative Breast Cancer
Source: PLoS One. 2012 Apr 18;7(4):e35569. doi: 10.1371/journal.pone.0035569 (PMC3329481; doi:10.1371/journal.pone.0035569)
Supplement: Text S1 — R code that describes the analyses performed with the supporting information data files. (PDF) [file pone.0035569.s001.pdf]

# Supplementary document for Kim et al. 2012

Ryung S. Kim

February 28, 2012

In this document, we provide the programming code to reproduce microarray analysis in Kim et al. 2012 using the statistical software R. The data files can be downloaded from http://ephpublic.aecom.yu.edu/sites/rkim/Supplementary/ .

```
library(survival)

dt.NKI295.signature <-read.delim("dt.NKI295.signature.xls",as.is=T,header=T)
dt.Neve.signature   <-read.delim("dt.Neve.signature.xls",as.is=T,header=T)
dt.Loi.signature    <-read.delim("dt.Loi.signature.xls",as.is=T,header=T)
dt.Pawitan.signature<-read.delim("dt.Pawitan.signature.xls",as.is=T,header=T)
dt.Wang.signature   <-read.delim("dt.Wang.signature.xls",as.is=T,header=T)

si        <- read.delim("si.xls",as.is=T,header=T,na.string=c(NA,""))
si$DS     <- factor(si$DS,levels=c("LDS","MDS","HDS"))
patients <- si$studytype=="patients"

#####FUNCTIONS FOR THE ANALYSIS #######
score.f <- function(dt.signature,values,is.cDNA=FALSE){
 if(is.cDNA) {
  scaled.intensity <- 10^dt.signature[,values]
 } else {
  scaled.intensity <- dt.signature[,values]/rowMeans(dt.signature[,values])
 }
 score<-colSums(log2(scaled.intensity[dt.signature$dormancy==1,]),na.rm=T) -
        colSums(log2(scaled.intensity[dt.signature$dormancy==-1,]),na.rm=T)
 return(data.frame(array=I(names(dt.signature)[values]),score=score))
}

discretize<-function(x,no.levels=3,labels=c("LDS","MDS","HDS")){
factor(
 colSums(sapply(x,function(y){y>quantile(x,(1:(no.levels))/no.levels,na.rm=T)})),
 labels=labels)
}

wtest.f<-function(dt,varname="ER",levels=c("+","-")){
 x<-dt$score[dt[,varname]==levels[1] & !is.na(dt[,varname])]
 y<-dt$score[dt[,varname]==levels[2] & !is.na(dt[,varname])]
 res<-data.frame(p.value=wilcox.test(x,y)$p.value,
   Median.DS.Group1=median(x,na.rm=T),
   Median.DS.Group2=median(y))
 names(res)<-gsub("1",levels[1],names(res))
 names(res)<-gsub("2",levels[2],names(res))
 names(res)<-gsub("Group",varname,names(res))
 return(res)
}

coxanalysis.f <- function(dt,stratified=F){
 DS1 <- DS2 <-dt$DS
```

```
 DS2[DS2=="HDS"]<-"LDS";DS2 <- as.factor(as.character(DS2))
 if(stratified){
      fit <- coxph(Surv(time.to.metastasis,metastasis)~DS1+strata(study), data=dt)
   rm.fit <- coxph(Surv(time.to.metastasis,metastasis)~DS2+strata(study), data=dt)
 } else {
      fit <- coxph(Surv(time.to.metastasis,metastasis)~DS1, data=dt)
   rm.fit <- coxph(Surv(time.to.metastasis,metastasis)~DS2, data=dt)
 }
 pvalue <-  anova(fit,rm.fit)[2,"P(>|Chi|)"]
 summary.fit <- summary(fit)
 hr<- summary.fit$conf.int[2,1]
 if(hr>1) {hr<-hr;conf.int<-summary.fit$conf.int[2,3:4]} else {
           hr<- - 1/hr;conf.int<- - 1/summary.fit$conf.int[2,4:3]}
 conf.int<-paste("(",paste(round(conf.int,3),collapse=" ,"),")",sep="")
 res<-data.frame(n=summary.fit$n,LRT.pvalue=pvalue,hazard.ratio=hr,
                 CI.95=I(conf.int))
 rownames(res)<-NULL
 return(res)
}

plot.f<-function(dt,study,plotid){
 for(i in 1:2){
  dt1<-dt[dt$ER == c("+","-")[i] & !is.na(dt$ER),]
  fit<-survfit(Surv(time.to.metastasis,metastasis)~DS,data=dt1)
  plot(fit,lty=2:4,ylab="Metastasis-Free Proportion",xlab="Years", col=1:3,
     main=paste(c("ER positive","ER negative")[i]," (n=",nrow(dt1),")\n
",study,sep=""),
     xlim=c(0,15))
  legend("bottomleft",
         c("Low-Dormancy Score","Mid-dormancy score","High-Dormancy Score"),
         lty = 2:4,col=1:3)
  mtext(plotid[i],cex=2,side=3,line=1,at=c(1))
 }
}

####Computation of dormancy scores######################
score.Loi     <- score.f(dt.Loi.signature,-(1:3))
score.Wang    <- score.f(dt.Wang.signature,-(1:3))
score.Pawitan <- score.f(dt.Pawitan.signature,-(1:3))
score.Neve    <- score.f(dt.Neve.signature,-(1:3))
score.NKI295  <- score.f(dt.NKI295.signature,-(1:3),is.cDNA=TRUE)

####Discretization of dormancy scores within each study and ER type #########
discretized.dormancy.scores <- by(si,list(si$study,si$ER),function(x){
                       data.frame(array=x$array,DS=discretize(x$score))
                       })

####Table 2#####################
print("Table 2: Do ER+ tumors have significantly higher dormancy scores than ER-
tumors? (Wilcoxon rank sum test)")

[1] "Table 2: Do ER+ tumors have significantly higher dormancy scores than ER- tumors?
(Wilcoxon rank sum test)"

by(si[patients,],si$study[patients],wtest.f)

si$study[patients]: Loi
    p.value Median.DS.ER+ Median.DS.ER-
1 0.8223647      1.552929      1.731837
-------------------------------------------------------------------------------------
si$study[patients]: NKI295
       p.value Median.DS.ER+ Median.DS.ER-
```

```
1 3.036319e-13      5.476198     -4.959639
------------------------------------------------------------------------------------
si$study[patients]: Pawitan
       p.value Median.DS.ER+ Median.DS.ER-
1 0.0008026373      2.512950     -3.105849
------------------------------------------------------------------------------------
si$study[patients]: Wang
       p.value Median.DS.ER+ Median.DS.ER-
1 1.623105e-09      3.432108     -4.866727


print("Van Elteren's test in Table 2")

[1] "Van Elteren's test in Table 2"

meta.dt <- si[si$studytype=="patients" & !is.na(si$ER),c("ER","score","study")]
meta.dt$ER <- (meta.dt$ER=="+")*1 + 2*(meta.dt$ER=="-")
write.table(meta.dt,"metadt.xls",sep="\t",row.names=F,quote=F,na="")
### STATA code
#clear
#cd C:\research\segall\
#insheet using metadt.xls, clear
#vanelteren score, by(er) strata(study)
#display r(p)
#exit

####Table 3#####################
print("Table 3: Compare metastasis-free survival between low/high dormancy groups (HR
and pvalue by Cox's PH model)")

[1] "Table 3: Compare metastasis-free survival between low/high dormancy groups (HR
and pvalue by Cox's PH model)"

by(si[patients,],list(si$study[patients],si$ER[patients]),coxanalysis.f)

: Loi
: -
   n LRT.pvalue hazard.ratio           CI.95
1 39  0.2775243     2.486488 (0.453 ,13.634)
------------------------------------------------------------------------------------
: NKI295
: -
   n LRT.pvalue hazard.ratio          CI.95
1 69  0.4163991     1.445947 (0.589 ,3.548)
------------------------------------------------------------------------------------
: Pawitan
: -
   n LRT.pvalue hazard.ratio            CI.95
1 29  0.2277022     -2.29576 (-0.569 ,-9.263)
------------------------------------------------------------------------------------
: Wang
: -
   n LRT.pvalue hazard.ratio            CI.95
1 77   0.999873    -1.000077 (-0.386 ,-2.593)
------------------------------------------------------------------------------------
: Loi
: +
    n LRT.pvalue hazard.ratio            CI.95
1 248 0.04968528    -1.940671 (-0.991 ,-3.802)
------------------------------------------------------------------------------------
: NKI295
: +
```

```
    n  LRT.pvalue hazard.ratio            CI.95
1 226 0.001023389    -2.624274 (-1.443 ,-4.773)
--------------------------------------------------------------------------------
: Pawitan
: +
    n LRT.pvalue hazard.ratio           CI.95
1 130 0.01386320    -3.309479 (-1.19 ,-9.201)
--------------------------------------------------------------------------------
: Wang
: +
    n LRT.pvalue hazard.ratio           CI.95
1 209 0.05237855    -1.655785 (-0.99 ,-2.768)

print("Meta analysis of four datasets using proportional hazards model stratified by
studies")

[1] "Meta analysis of four datasets using proportional hazards model stratified by
studies"

by(si[patients,],si$ER[patients],coxanalysis.f,T)

si$ER[patients]: -
    n LRT.pvalue hazard.ratio          CI.95
1 214  0.6946387     1.115683 (0.646 ,1.928)
--------------------------------------------------------------------------------
si$ER[patients]: +
    n   LRT.pvalue hazard.ratio           CI.95
1 813 2.366943e-06    -2.116641 (-1.539 ,-2.91)

####From Results section#############
print("... 51 breast cancer cell lines grown in tissue culture (8). We found that ER
positive breast cancer cell lines have significantly higher dormancy scores than ER
negative ones...")

[1] "... 51 breast cancer cell lines grown in tissue culture (8). We found that ER
positive breast cancer cell lines have significantly higher dormancy scores than ER
negative ones..."

wtest.f(si[si$study=="Neve",])

       p.value Median.DS.ER+ Median.DS.ER-
1 9.054645e-05      11.08920     -3.723333

print("We found that there was no statistically significant correlation between
proliferation index and dormancy score for ERcell lines (p>.9), but a significant
correlation for ER+ cell lines (Spearman correlation coefficient -0.76, p<.01, Figure
1B).")

[1] "We found that there was no statistically significant correlation between
proliferation index and dormancy score for ERcell lines (p>.9), but a significant
correlation for ER+ cell lines (Spearman correlation coefficient -0.76, p<.01, Figure
1B)."

pi.dt<-si[!is.na(si$score)  & !is.na(si$proliferation.index.3d.percent),]
pi.dt<-pi.dt[pi.dt$array!="HCC1500",]
x<-pi.dt$score;y<-pi.dt$proliferation.index.3d.percent
index<-pi.dt$ER=="+"
cor.test(x[index],y[index],method="spearman")

        Spearman's rank correlation rho

data:  x[index] and y[index]
```

```
S = 387.3808, p-value = 0.006545
alternative hypothesis: true rho is not equal to 0
sample estimates:
      rho
-0.760822

Warning message:
In cor.test.default(x[index], y[index], method = "spearman") :
  Cannot compute exact p-values with ties

cor.test(x[!index],y[!index],method="spearman")

        Spearman's rank correlation rho

data:  x[!index] and y[!index]
S = 214, p-value = 0.946
alternative hypothesis: true rho is not equal to 0
sample estimates:
       rho
0.02727273

####From Discussion section##########
si.temp<-si[si$subtype %in% c("LuminalA","LuminalB") & !is.na(si$subtype),]
print("...the median dormancy score is significantly lower in the luminal B tumors
compared to the luminal A tumors...(Wilcoxon rank sum test)")

[1] "...the median dormancy score is significantly lower in the luminal B tumors
compared to the luminal A tumors...(Wilcoxon rank sum test)"

by(si.temp,si.temp$study,wtest.f,"subtype",c("LuminalA","LuminalB"))

si.temp$study: NKI295
       p.value Median.DS.subtypeLuminalA Median.DS.subtypeLuminalB
1 4.266688e-05                  6.799987                  1.077966
--------------------------------------------------------------------------------------
si.temp$study: Pawitan
       p.value Median.DS.subtypeLuminalA Median.DS.subtypeLuminalB
1 6.711862e-06                  2.342841                 -2.510712

print("...in a metaanalysis of all luminal B tumors from the two datasets, we found
2.6 times lower hazard of metastasis among patients with high dormancy score tumors
compared to those with low dormancy scores (p<.04)")

[1] "...in a metaanalysis of all luminal B tumors from the two datasets, we found 2.6
times lower hazard of metastasis among patients with high dormancy score tumors
compared to those with low dormancy scores (p<.04)"

by(si.temp,si.temp$subtype,coxanalysis.f,T)

si.temp$subtype: LuminalA
    n LRT.pvalue hazard.ratio            CI.95
1 127   0.638882    -1.269938 (-0.471 ,-3.425)
--------------------------------------------------------------------------------------
si.temp$subtype: LuminalB
    n LRT.pvalue hazard.ratio            CI.95
1 104 0.03614516    -2.590109 (-0.978 ,-6.863)

####Figure 1B####################
tiff(filename = "proliferation.index.tif", width = 360, height = 360)
par(lwd=2);par(bty="l");par(cex.axis=1.5);par(cex.lab=1.5)
plot(x,y,ylab="Proliferation Index",xlab="Dormancy Score",type="n",ylim=c(0,100))
points(x[index],y[index],col="skyblue",lty=1)
```

```
abline(lm(y[index]~x[index])$coef,col="skyblue")
points(x[!index],y[!index],col="violet",lty=2)
abline(lm(y[!index]~x[!index])$coef,col="violet")
dev.off()

####Figure 2####################
tiff(filename = "figure2.tif", width = 720, height = 360*3)
par(mfrow=c(4,2))
par(lwd=2);par(bty="l");par(cex.axis=1.5);par(cex.lab=1.5)
plot.f(si[si$study=="NKI295",],"Van de Vijver et al.",c("A","B"))
plot.f(si[si$study=="Loi",],"Loi et al.",c("C","D"))
plot.f(si[si$study=="Wang",],"Wang et al.",c("E","F"))
plot.f(si[si$study=="Pawitan",],"Pawitan et al.",c("G","H"))
dev.off()
```

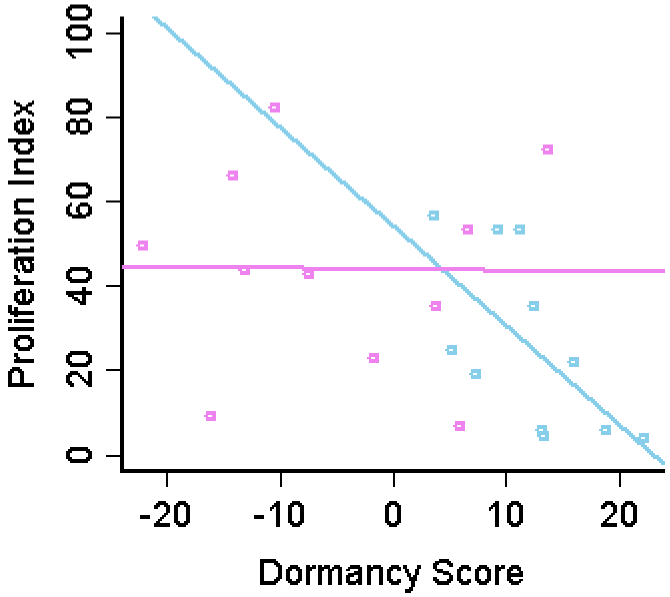


<Figure 1B>

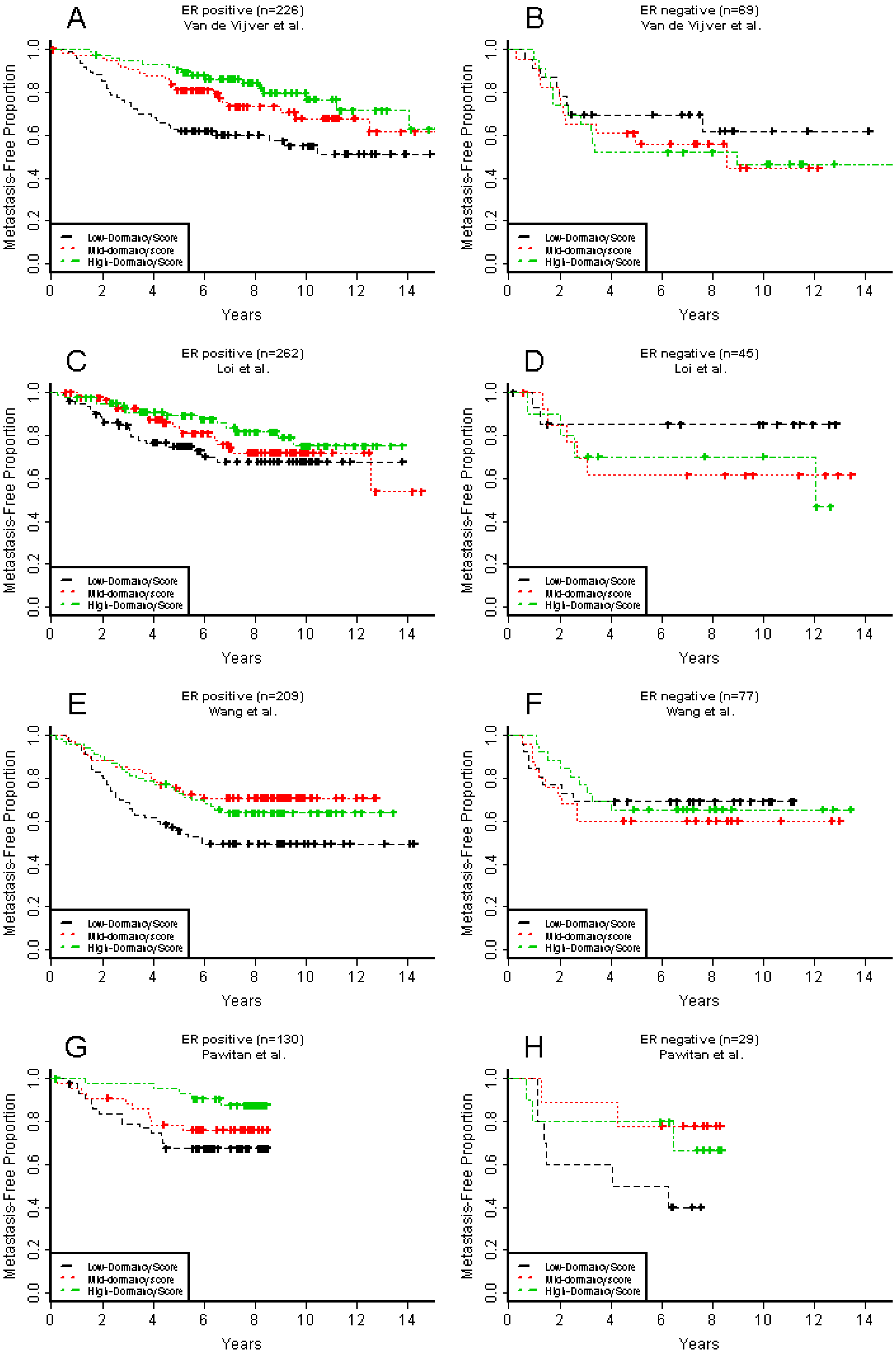
A
ER positive (n=226)
Van de Vijver et al.
B
ER negative (n=69)
Van de Vijver et al.
C
ER positive (n=262)
Loi et al.
D
ER negative (n=45)
Loi et al.
E
ER positive (n=209)
Wang et al.
F
ER negative (n=77)
Wang et al.
G
ER positive (n=130)
Pawitan et al.
H
ER negative (n=29)
Pawitan et al.
Metastasis-Free Proportion
Years
0.0
0.2
0.4
0.6
0.8
1.0
0
2
4
6
8
10
12
14

<Figure 2>
